# Enrichment in the Sucker and Weaner Phase Altered the Performance of Pigs in Three Behavioural Tests

**DOI:** 10.3390/ani8050074

**Published:** 2018-05-14

**Authors:** Cameron Ralph, Michelle Hebart, Greg M. Cronin

**Affiliations:** 1 Animal Welfare Science Centre, South Australian Research and Development Institute, Roseworthy Campus, Roseworthy, SA 5371, Australia; 2 Animal Welfare Science Centre, School of Animal and Veterinary Sciences, The University of Adelaide, Roseworthy, SA 5371, Australia; michelle.hebart@adelaide.edu.au; 3 Faculty of Science, School of Life and Environmental Sciences, University of Sydney, 425 Werombi Road, Camden, NSW 2570, Australia; greg.cronin@sydney.edu.au

**Keywords:** enrichment block, early life experience, pig welfare

## Abstract

**Simple Summary:**

Two important questions about the intensive housing of pigs concern whether young pigs require environmental enrichment to enhance their welfare, and from what age should enrichment be provided to pigs to achieve the benefit? We provided sucker and weaner pigs with enrichment in the form of blocks, which they could push around the floor of the pen, compared to no blocks (‘barren’ control). Behavioural and physiological responses of the pigs were then measured in a series of standard tests used to assess fear response, learning, and cognitive ability. Enrichment blocks modified the behavioural responses of pigs in the different tests, suggesting enriched pigs were more willing to explore novel environments and they had increased ability to learn. However, our enrichment treatment did not alter the pigs’ cortisol response, suggesting any differences due to enrichment were subtle. In contrast, the altered behavioural responses probably indicate that although pigs readily learned complex tasks and modify their behaviour to suit the current situation, there may be some potential benefits from enrichment when applied during the early weeks of a pig’s life that might have life-long benefits for the animal and its welfare.

**Abstract:**

We tested the hypothesis that provision of enrichment in the form of enrichment blocks during the sucker and weaner phases would affect the behaviour of pigs. We measured the performance of pigs in an open field/novel object test, a maze test, an executive function test and the cortisol response of the pigs after exposure to an open field test. The provision of enrichment blocks altered the behaviour of the pigs in all three tests and these changes suggest an increased willingness to explore and possibly an increased ability to learn. The behavioural tests highlighted that young pigs have the capacity to learn complex tasks. Our findings support the notion that the benefits of enrichment cannot be evaluated by measuring the interactions the animal has with the enrichments in the home pen and it may simply be beneficial to live in a more complex environment. We have highlighted that the early rearing environment is important and that the management and husbandry at an early age can have long-term implications for pigs. The enrichment we used in this study was very simple, an enrichment block, and we provide evidence suggesting the provision of enrichment effected pig behavioural responses. Even the simplest of enrichments may have benefits for the welfare and development of young pigs and there is merit in developing enrichment devices that are suitable for use in pig production.

## 1. Introduction

Environmental enrichment is the modification of a barren captive-environment to improve the biological functioning of animals [1]. Enrichments can enhance the welfare of animals by allowing them to perform more of their species-specific behavioural repertoire and accommodate a greater range of behavioural choices [2]. Enrichments generally provide novelty, social contact, and exercise that is rewarding and result in net overall benefit for the animal [1]. Whilst environmental enrichment is accepted to improve the welfare of animals, animals housed in enriched environments have greater glucocorticoid concentrations than animals housed in barren environments [3]. Therefore, assessing the welfare benefits of environmental enrichment poses a number of challenges.

Evaluating the benefit of environmental enrichment is challenging, however, animals housed in enriched environments can be less anxious, engage in more social contact, cope better with stress, and have stronger immune systems. Meehan and Mench [4] referred to environmental enrichment as a positive stressor and acknowledged that the lack of challenge in an environment is as detrimental to welfare as too much challenge. Moreover, when challenge is applied in a species-specific and appropriate manner, the welfare of the animal is improved. In 1976, Selye introduced the concept of eustress and distress with distress being stress that has a negative effect and eustress being stress that has a positive effect [5]. Selye described how eustress would enable adaptation and make an animal better able to cope whilst distress would lead to a pathological state. Environmental enrichment could be viewed as eustress in that it enables adaptation and by doing so equips animals to cope better with subsequent stressors. Crofton et al. [1] described environmental enrichment as inoculation stress. The latter is a process by which animals develop resilience to future stressful experiences by first being exposed to mildly stressful events early in life [1,6]. Evidence in a range of species supports this and indicates that the best way to evaluate the benefit of environmental enrichment is to test how animals in enriched environments cope with experiences outside of their home pen.

Laboratory mice raised in enriched cages had significantly greater basal corticosterone concentrations than mice raised in barren cages [3]. When exposed to a stress paradigm, elevated plus maze, and staircase, mice from enriched cages showed no significant increase in plasma corticosterone whereas mice from barren cages did show a significant increase in corticosterone [3]. Mice from enriched cages explored the maze more, were more active, and had greater natural killer cell activity than controls [3]. In a similar study, it was shown that neonatal handling enabled adult mice to cope better with a swim test [7]. Pigs that were kept on deep litter had greater 24 h salivary cortisol than pigs that were kept in barren cages and this has been shown in a number of experiments [8,9]. In addition, pigs exposed to an enriched rewarding environment containing straw and chocolate raisins had a significant increase in cortisol that was very similar to the cortisol response of pigs exposed to an aversive environment consisting of a barren concrete floor and intermittent snout roping [10]. Pigs housed in barren conditions had greater cortisol responses to transport, handling, and lairage than pigs housed on straw [8,9,11]. Pigs housed in barren environments experienced greater cortisol responses at slaughter than pigs housed in enriched environments [9].

Environmental enrichment enhances the welfare of animals and activates the hypothalamic-pituitary adrenal (HPA axis) and the sympathoadrenal system whilst the brains of animals housed in enriched environments undergo molecular and morphological changes that lead to enhanced learning, memory, and ability to cope with stress [12,13]. Assessing the value of environmental enrichment is challenging and we propose that the main benefit of enrichment to the welfare of pigs is an enhanced ability to learn and enhanced ability to cope with stressors. This has multiple benefits for the pig and for the pig industry because if appropriate enrichment is provided animals may be more resilient, be less susceptible to disease, and be easier to move and to handle in addition to enrichments having other potential benefits for pig welfare. There is little research published on the benefits of enrichment to pigs in this context. We tested the hypothesis that the provision of enrichment in the form of enrichment blocks during the sucker and weaner phases would affect the behaviour of pigs.

## 2. Materials and Methods

**Figure 1:**
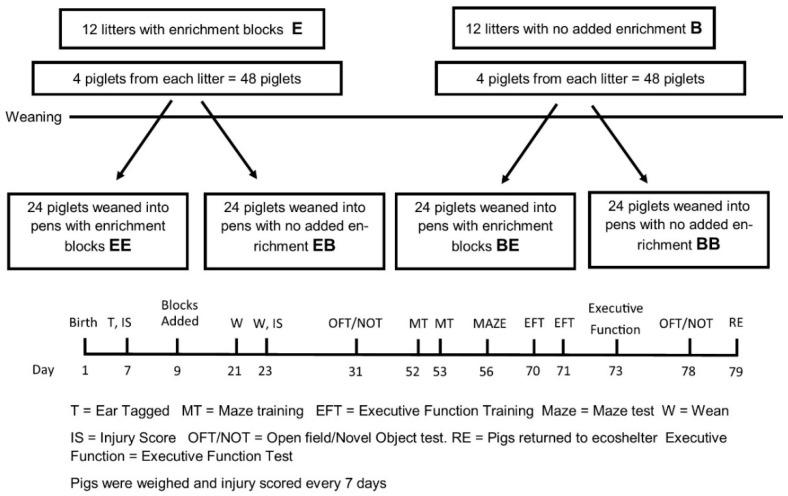
The experimental design for the experiment. The top panel depicts the provision of enrichment in the sucker and weaner phases and the 2 × 2 factorial design. The bottom panel shows the time line of events in each replicate of the experiment.

All procedures were approved by the Department of Primary Industries and Regions South Australia Animal Ethics Committee (AEC project number 34/15). Piglets (Large White × Landrace) were housed in conventional farrowing crates for 21 days during lactation and then in group (weaner) pens until 11 weeks of age. Litters (*n* = 96) were randomly assigned to treatments within a 2 × 2 factorial design, with four replicates in time. Replicate one ran from February to April 2017, replicate two ran from September to November 2017, replicate three ran from November 2017 to February 2018, and replicate four ran from February 2018 to April 2018. In each replicate, four focal pigs were selected from each litter (two males and two females) for experimental observation. Therefore, there were 24 pigs per treatment in each replicate selected from 24 litters. The pigs were raised with either enrichment (E) or no enrichment (barren: B) provided for the crate/pen, with cross-over between the sucker and weaner phases. At weaning, the four focal pigs per litter were grouped in weaner pens with a total of 24 weaners per replicate of the same treatment grouping (See Figure 1). Thus, there were four treatments: enriched in sucker phase and enriched in weaner phase (EE; *n* = 96), enriched in the sucker phase and barren in the weaner phase (EB; *n* = 96), barren in the sucker phase and enriched the weaner phase (BE; *n* = 96), and barren in the sucker phase and barren in the weaner phase (BB; *n* = 96). The experimental unit was the individual pig. Food and water were provided ad libitum during the weaner phase. One block per four pigs was provided, that is, a pen of 24 pigs had six blocks and a farrowing crate with 12 piglets would have three blocks. Enrichment blocks were replaced weekly and the cubic-shaped blocks provided were increased in size between the sucker and weaner stage to match piglet size. The enrichment blocks were a “nutritional lick block” developed by Ridley Agriproducts (Toowong, Queensland, Australia) and were specifically formulated for use with sucker and weaner pigs [14].

A series of tests were conducted throughout the 11 weeks and these are described in detail below. Pigs were weighed weekly and were physically assessed weekly throughout the experiment, with the number of scratches on the integument counted and live weight recorded. Pigs were also scratch- and injury-scored one day after weaning. At day 31 of life, 6 pigs per treatment per replicate (i.e., 24 pigs total per treatment over the 4 replicates in time) were exposed to an open field/novel object test. Similarly, at day 56 of life a second set of 6 pigs per replicate (24 pigs total) were exposed to a maze test, and at day 73 of life a third set of 6 pigs per replicate (24 pigs total) were exposed to an executive function test. Finally, at day 78 of life the remaining 6 pigs per treatment per replicate were exposed to an open field/novel object test where blood samples were collected every 15 min for 2 h prior to the test and every 15 min for 2 h after the test. The experiment was designed such that each pig was only exposed to one test; no pigs were exposed to multiple tests.

### 2.1. Open Field/Novel Object Test

**Figure 2:**
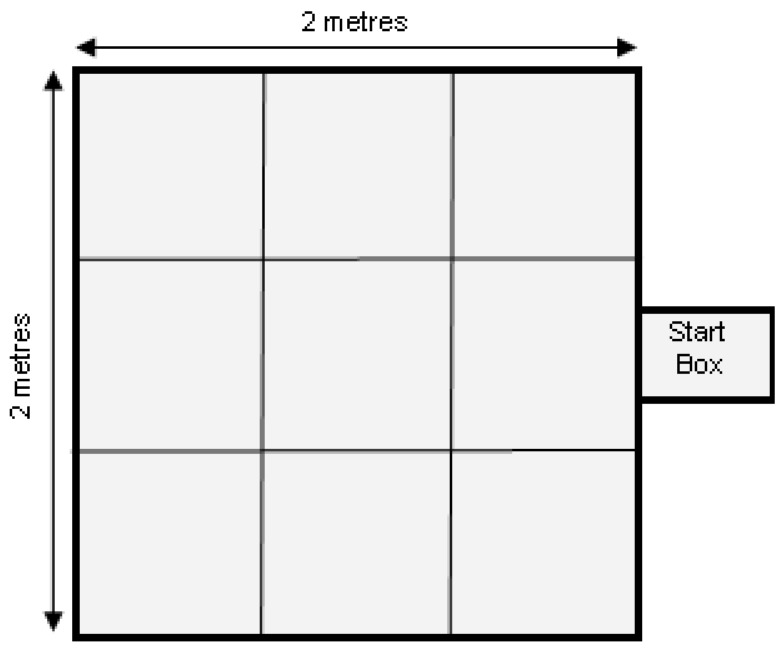
Test arena for Open Field and Novel Object Test.

On day 31 of life, 6 pigs per replicate from each treatment (totalling 24 pigs per treatment over the 4 replicates in time) were exposed to an open field test/novel object test. Figure 2 depicts the testing apparatus. The piglet was placed inside the start box and after 1 min was provided the opportunity to emerge from the start box into the arena. Emergence time from the start box was recorded and then behaviours listed in Table 1 were recorded in real time. After 3 min in the arena, a novel object (red bucket) was introduced (from above, lowered on a rope) and the piglet remained in the arena for a further 2 min. At the conclusion of the test, the piglet was returned to its home pen.

### 2.2. Maze Test

**Figure 3:**
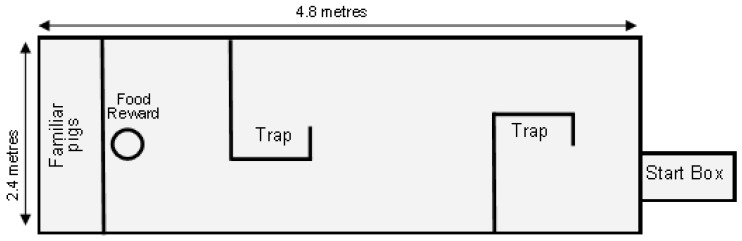
Test arena for the Maze Test.

At 56 days of life, 6 pigs per replicate, 24 pigs in total per time replicate, were individually exposed to a maze test. The maze testing apparatus is shown in Figure 3. The test consisted of two phases: the training phase (conducted on days 52 and 53 of life) and the testing phase (conducted on day 56 of life).

#### Training Phase

Each group of six pigs was introduced to the arena via the start box on two occasions for 5 min before the maze was installed (pre-training phase). Two pigs familiar to the test pigs (i.e., non-experimental pigs from their home pen) were present in the pen, at the opposite end to the start box (Figure 3). Each pig was then individually exposed to three training runs in the arena without the maze, then two days later were exposed to four test runs with the maze in place. The maze was constructed from wire mesh panels which allowed the test pig to see the opposite end of the arena, where two familiar pigs were penned as lures. Figure 3 shows the plan of the completed test arena, consisting of two traps, a start box at one end of the maze, and, at the opposite end, two familiar pigs and the reward (canned cream in a bowl). The start box had a transparent door enabling the pigs to see through the maze while standing in the start box. Each pig was held in the start box for 1 min and then released into the maze. Researchers recorded time to exit the start box, time taken to navigate through the maze and reach the reward, the number of times that the pig entered a trap, and the total time spent in the trap (Table 2). The difference in time taken to complete the maze between test 1 and test 4 was also evaluated.

### 2.3. Executive Function Test

**Figure 4:**
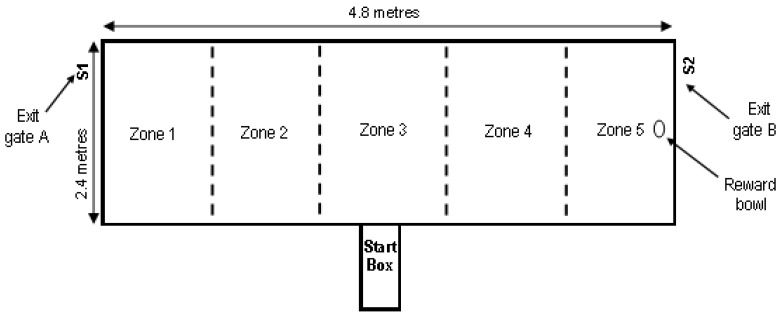
Test arena for Executive Function Test. S1 depicts speaker 1 and S2 depicts speaker 2. Reward bowl changed ends depending on the audio que.

The executive function test assessed the ability of the animal to learn an audible cue and associate that cue with a food reward. The arena used in the executive function test is shown in Figure 4. The test consisted of two phases: the training phase and the testing phase.

**Training phase: (1) habituation of the group of focal pigs to the arena**—The pigs were introduced to the arena as a group of six through the start box, with the order of entry of the treatment groups re-randomized for each time replicate. The start box entry and exit doors were open to allow the six pigs to move into the arena without hindrance; after the last pig entered the arena the exit door was closed to prevent pigs leaving the arena via the start box. The group remained in the test arena for 5 min and the pigs were allowed to freely explore the arena. The group of six pigs was then quietly moved out of the test arena via one of the two exit gates, before being re-introduced to the test arena through the start box and again allowed 5 min to freely explore the test arena. The pigs were then quietly moved out of the test arena via the other exit gate. Four groups of pigs were trained each day. Groups one and three exited from gate A first, while groups two and four exited from gate B first. This ensured we avoided introducing a bias to the pigs by only removing them through one exit gate.

**Training phase: (2) habituation of individual focal pigs to the procedure**—The aim of this phase was to determine the natural inclination of the pig to turn left or right after exiting the start box, and to condition the pig to associate a sound with a food reward.

A food reward was placed at both ends of the arena. The pig was held in the start box for 10 s (s) and the sounds became audible to the pig after 8 s in the start box, i.e., 2 s prior to being released from the start box. For three focal pigs per treatment group, sound A was broadcast from speaker 1 and sound B was broadcast from speaker 2 (Figure 4). Both sounds were broadcast at the same time. For the other three focal pigs in the group, the positions of the sounds were reversed.

Time taken to exit the start box, movement in the arena based on number of zones entered, and end time identified when the pig crossed either end zone line (i.e., entered zone 1 or 5 (Figure 4)) or made contact with the bowl containing the food reward, were recorded. Also recorded was which end of the arena the pig first moved towards after exiting the start box, and which end of the arena was reached first (the end line/food reward). Once the pig reached one end of the test arena it was allowed to exit the arena via the exit gate at that end. Each pig was exposed to this procedure three times on training day 1 and four times on training day 2.

This training phase enabled the researchers to determine which side of the arena the pig was naturally inclined to turn towards (i.e., side-bias—if the pigs preferred the left side or the right side) and train the pig to associate the food reward with the sound located at the end of the arena that the pig preferred (the pig’s preferred sound). This information was recorded and was critical to the testing phase.

#### Testing Procedure—Executive Function Test

Each focal pig was tested individually three times across the testing day. The pig was held in the start box for 10 s and the sounds became audible to the pig after 8 s in the start box, i.e., 2 s prior to being released from the start box.

During the test the pigs were presented with both sounds and the sound that indicated food reward was broadcast from the opposite side to their previously determined preference. The preferred sound for the respective pig was presented on the pig’s non-preferred side and the food reward was only available at the side of the arena where the preferred sound was located. For example, if it was determined during the training phase that the pig preferred side A and sound 1 then during the testing phase sound 1 and the food reward would be placed at side B. The time taken to exit the start box and movement in the arena until the pig crossed either the end zone line (i.e., enters zone 1 or 5 in Figure 4) or made contact with either the bowl containing the food reward or the bowl without the food reward was recorded (Table 3). If none of these occurred the test was terminated after 2 min.

At the end of each test the test pig was quietly removed from the end where the cream was located.

### 2.4. Cortisol Response to Novel Object/Open Field Test

This test was applied as described in Section 2.1 above. Indwelling ear vein catheters were implanted in the pigs at 10:00 in the morning of the test and the pigs were given 4 h to recover from the procedure. Blood samples were collected 120 min, 90 min, 60 min, 30 min, 15 min, and 1 min prior to the test and then 15 min, 30 min, 60 min, 90 min, and 120 min after the completion of the open field test. Blood samples were centrifuged at 3000 rpm for 10 min, with plasma harvested and frozen at −20 °C until analysis. Plasma was assayed for cortisol using radio-immuno assay (MP Biomedicals, Santa Ana, CA, USA).

### 2.5. Statistical Analysis

All data were analysed using a mixed model in ASReml version 4.1. (VSN International Ltd., Hemel Hempstead, UK). Any continuous data that were not normally distributed were transformed (either log or square root). All binary data were analysed using a generalised linear mixed model with the logit-link function, where the implicit residual variance on the underlying scale is π2/3. Count data were analysed assuming a Poisson distribution or where there was over-dispersion a negative binomial regression.

The fixed effects fitted to the executive function and novel object data included replicate (1–4), test (1–3), parity (0–5), sex (F, M), sucker enrichment (barren or enriched), weaner enrichment (barren or enriched), and all significant (*p* < 0.05) two-way interactions. To account for repeated measures on animals, animal ID was included as a random term. Sow ID was also fitted as a random term to separate the within and between litter variation. The same model was fitted to the maze data but the fixed effect of round (1–4) was included rather than test.

Three analyses were conducted for the open field/novel object test. Data for the open field test (first 3 min) were considered separately to the data from the novel object test (last 2 min). Data were then analysed for the entire 5 min test period. Non-normally distributed data were transformed using natural-logarithms and when this occurred back-transformed means are shown in parenthesis. The fixed effects included in the model were replicate (1–4), parity (0–5), sex (F, M), sucker enrichment (barren or enriched), weaner enrichment (barren or enriched), and all significant (*p* < 0.05) two-way interactions. There were no repeated measures on this data so only Sow ID was included as a random term.

The cortisol data were tested for normality using the Kolmogorov-Smirnov statistic and homogeneity of variance was tested using Levene’s test. No transformations were necessary. Repeated measures analysis of variance (ANOVA) was used to compare the plasma concentrations of cortisol within and between groups. The within-subjects factor was time and the between-subjects factor was treatment. Scratch scores and live weight data were analysed using a mixed model with treatment, phase, and week as fixed effects and ID was included as a random term (to account for repeated measures).

## 3. Results

### 3.1. Open Field/Novel Object Test

**Figure 5:**
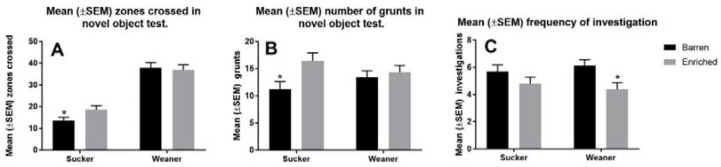
Behavioural responses of pigs in the novel object test. Panel (**A**) shows the number of lines crossed (zones entered), panel (**B**) shows the number of grunts, and panel (**C**) shows the number of investigations of the novel object. * indicates a significant difference (*p* < 0.05) between pigs provided with enrichment and pigs that were housed in a barren environment in each phase.

The mean (±SEM) number of lines crossed during the novel object test was significantly greater for pigs provided with enrichment during the sucker phase than pigs housed in barren pens during the sucker phase (18.66 ± 1.82 vs. 13.52 ± 1.63, *p* < 0.01, Figure 5). The mean (±SEM) number of grunts produced during the novel object test was greater for pigs provided with enrichment during the sucker phase than pigs housed in barren pens during the sucker phase (16.46 ± 1.43 vs. 11.30 ± 1.33, *p* < 0.01). The mean (±SEM) number of times that the pig investigated the novel object was significantly greater for pigs raised in barren pens in the weaner phase than pigs that were provided with enrichment in the weaner phase (6.12 ± 0.48 vs. 4.40 ± 0.46, *p* < 0.01). There was no significant effect of enrichment on emergence time, number of grunts during the open field test, the number of lines crossed during the open field test, the time taken to interact with the novel object, or number of squeals during the open field test or the novel object test or the number of urinations.

### 3.2. Maze Test

**Figure 6:**
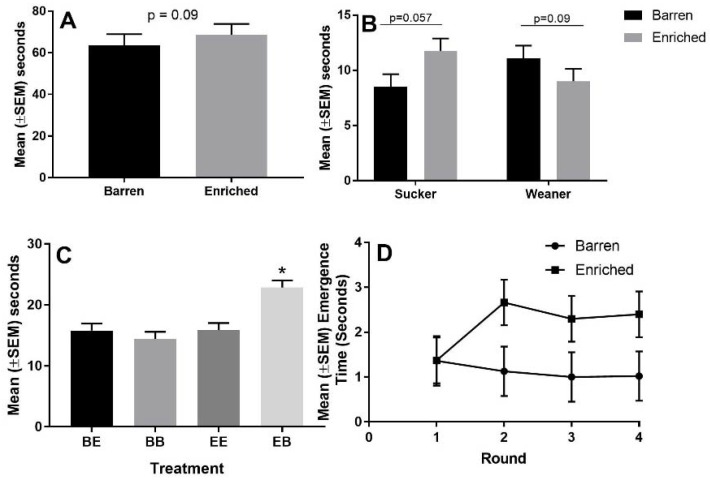
The effect of enrichment on performance in the maze test. Panel (**A**) depicts the mean time taken to reach the reward for pigs that were housed in enriched pens in the sucker phase or housed in barren pens in the sucker phase, panel (**B**) depicts mean (±SEM) time spent in trap 2, panel (**C**) depicts total time spent in all traps, and panel (**D**) depicts the time taken to emerge from the start box over the four rounds of the test. * indicates significant difference, *p* < 0.05.

In round one of the maze test, the mean (±SEM) time taken for pigs to emerge from the start box was not significantly different between pigs provided with enrichment in the sucker phase and pigs housed in barren pens during the sucker phase (1.35 ± 0.55 s vs. 1.37 ± 0.51 s, *p* > 0.05). In rounds two, three, and four, the mean (±SEM) time taken for pigs to emerge from the start box was significantly greater (*p* < 0.05) for pigs provided with enrichment during the sucker phase than pigs housed in barren environments during the sucker phase (Round 2: 2.65 ± 0.51 s vs. 1.13 ± 0.55 s; round 3: 2.3 ± 0.51 s vs. 1.00 ± 0.55 s; round 4: 2.4 ± 0.51 vs. 1.02 ± 0.51). The mean (±SEM) total time (s) spent in all traps was significantly greater for EB pigs than EE pigs, BE pigs, or BB pigs (22.86 ± 1.16 vs. 15.88 ± 1.16 vs. 14.12 ± 1.17 vs. 15.81 ± 1.16, *p* < 0.05). There was a trend (*p* = 0.09) toward the mean (±SEM) time taken to reach the reward being greater for pigs provided with enrichment in the sucker phase than pigs housed in a barren environment during the sucker phase (68.77 ± 5.16 vs. 63.66 ± 5.16, *p* = 0.09 Figure 6). There was a trend (*p* = 0.057) toward the mean (±SEM) time spent in trap two being greater for pigs enriched in the sucker phase than pigs housed in barren pens in the sucker phase (11.76 ± 1.12 s vs. 8.51 ± 1.12, *p* = 0.057). There was a trend (*p* = 0.09) toward the mean (±SEM) time spent in trap 2 to be less for pigs provided with enrichment in the weaner phase than pigs housed in barren pens in the weaner phase (9.01 ± 1.12 s vs. 11.11 ± 1.12 s).

**Figure 7:**
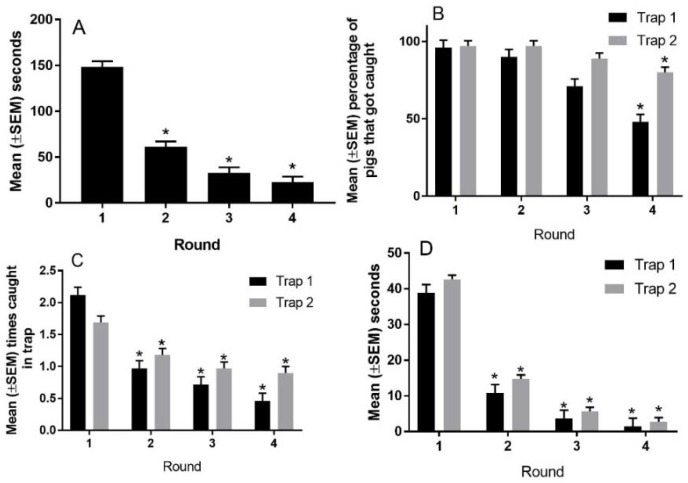
The performance of pigs in the maze test over successive rounds. Panel (**A**) depicts the time taken to solve the maze over four rounds for all pigs, panel (**B**) depicts the percentage of pigs that got caught in trap 1 or 2 over the four rounds for all pigs, panel (**C**) depicts the number of times a pig was caught in each trap for all pigs, and panel (**D**) depicts the amount of time pigs spent in each trap for all pigs. * indicates a significant difference from round one (*p* < 0.05).

When the performance of all pigs in the maze test was analysed there were significant effects of round on the time taken to solve the maze, the percentage of pigs that entered trap one, the percentage that entered trap two, the number of times pigs entered a trap, and the time spent in trap one and trap two over the four rounds (Figure 7). The mean (±SEM) time taken to solve the maze reduced from 148.4 ± 6.13 s in round one to 61.0 ± 6.11 s in round two, to 32.8 ± 6.06 s in round three to 22.7 ± 9.09 s in round four (*p* < 0.001). The mean (±SEM) percentage of pigs that entered trap one progressively declined with repeated testing, from 96 ± 0.04% in round one, to 90 ± 0.04% in round two, to 71 ± 0.04% in round three, to 48 ± 0.04% in round four (*p* < 0.001). The mean (±SEM) percentage of pigs that entered trap two in rounds one and two was 97 ± 0.03%, respectively. This proportion was reduced to 88 ± 0.03% in round three and 80 ± 0.3% in round four (*p* < 0.01). The mean (±SEM) number of times that pigs entered trap one reduced from 2.2 ± 0.12 in round one, to 1.0 ± 0.12 in round two, to 0.7 ± 0.12 in round three, to 0.5 ± 0.12 in round four (*p* < 0.001). The mean (±SEM) number of times that pigs entered trap two reduced from 1.7 ± 0.10 in round one, to 1.2 ± 0.10 in round two, to 1.0 ± 0.10 in round three, to 0.9 ± 0.10 in round four (*p* < 0.001). The mean (±SEM) total time that pigs spent in trap one reduced from 38.8 ± 2.34 s in round one, to 10.9 ± 2.34 s in round two, to 3.7 ± 2.34 in round three, to 1.4 ± 2.34 s in round four (*p* < 0.001). The mean (±SEM) total time that pigs spent in trap two reduced from 42.7 ± 1.13 s in round one, to 14.8 ± 1.13 s in round two, to 5.7 ± 1.13 in round three, to 2.8 ± 1.14 s in round four (*p* < 0.001).

### 3.3. Executive Function Test

**Figure 8:**
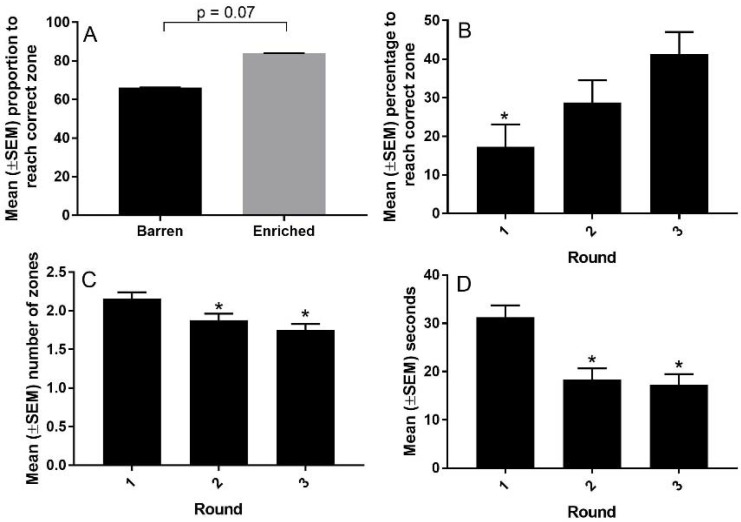
The effect of enrichment and round on the performance of pigs in the executive function test. Panel (**A**) shows that proportion of pigs that reached the reward when enriched in the weaner phase. Panel (**B**) depicts the percentage of all pigs that reached the reward for rounds 1, 2, and 3. Panel (**C**) depicts the mean number of zones that the pigs crossed before they reached the reward for rounds 1, 2, and 3. Panel (**D**) depicts the time taken to reach the reward for all pigs in rounds 1, 2, and 3. * = significant difference from round 1, *p* < 0.05.

There was a trend (*p* = 0.07) for an increase in the mean (±SEM) proportion of pigs enriched in the weaner phase that reached the correct zone than pigs housed in barren pens in the weaner phase (84 ± 0.08% vs. 66 ± 0.12%, Figure 8). There were no other significant effects of enrichment. There was a significant (*p* < 0.001) increase in the mean (±SEM) percentage of pigs that reached the correct zone on their first try, from 17 ± 0.06% in round one to 29% ± 0.06 in round two to 41% ± 0.06 in round three (Figure 8). There was a significant (*p* < 0.001) decrease in the number of zones crossed to get to the correct zone, from 2.1 ± 0.08 zones crossed in round one to 1.9 ± 0.08 zones crossed in round two to 1.8 ± 0.08 zones crossed in round three (Figure 8). There was a significant (*p* < 0.01) decrease in the mean (±SEM) time taken to reach the correct zone, from 31.3 ± 2.43 s in round one to 18.8 ± 2.23 s in round two to 17.3 ± 2.19 s in round three (Figure 8).

### 3.4. Cortisol Response to Novel Object/Open Field Test

**Figure 9:**
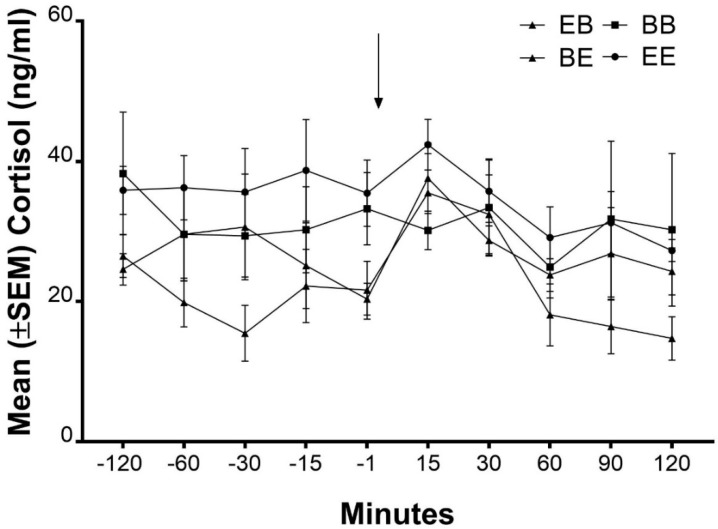
The mean (±SEM) change in cortisol before and after exposure to an open field test. Pigs were exposed to a 3 min open field test (indicated by the arrow). Blood samples were collected 120 min, 90 min, 60 min, 30 min, 15 min, and 1 min prior to the test and then 15 min, 30 min, 60 min, 90 min, and 120 min after the completion of the open field test.

There was no effect of treatment on the concentration of cortisol in plasma measured for 2 h prior to the introduction into an open field/novel object test or for 2 h after an open field/novel object test (Figure 9, *p* > 0.05).

### 3.5. Scratch Score and Body Weight

**Figure 10:**
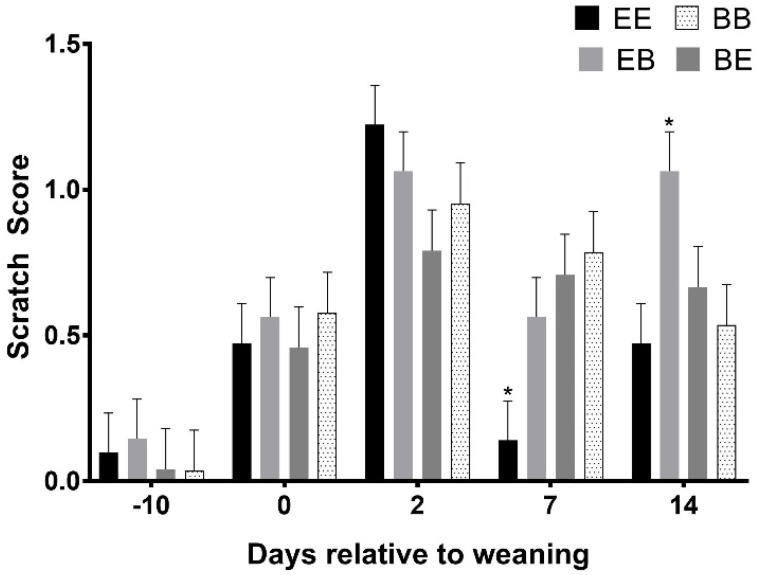
The effect of the provision of enrichment on the number of scratches (scratch score) recorded on the pigs. Day 0 indicates the day of weaning; other days are relative to weaning. * indicates a significant difference between the groups, *p* < 0.05.

**Figure 11:**
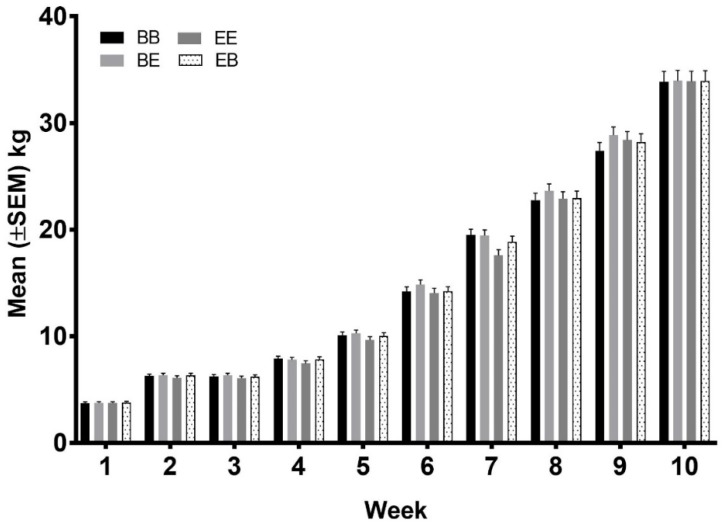
The effect of the provision of enrichment on pig weight to 10 weeks of age. Pigs were weighed weekly from week 1 to week 10.

There were significantly fewer scratches on pigs from the EE group 7 days after weaning than the other groups (*p* < 0.05) and significantly more scratches on the pigs in the EB group that the other groups 14 d after weaning (*p* < 0.05; Figure 10). There were no significant differences in the body weight of the pigs between treatments at any time (*p* > 0.05; Figure 11).

## 4. Discussion

Our data suggest that the provision of enrichment blocks affected the behaviour of pigs in an open field/novel object test, a maze test, and an executive function test. The performance of the pigs in the maze and executive function tests improved significantly with each exposure to the respective tests and this is a good indication that the pigs demonstrated learning. The reduced number of scratches on pigs that were enriched in both the sucker and the weaner phase 7 days after weaning suggests that there were possibly effects on aggression (and fighting) and that the provision of enrichment may have reduced the number or severity of the fights. The greater number of scratches on pigs that were enriched in the sucker phase, but not in the weaner phase 14 days after weaning, suggests there may have been more fights or more aggressive encounters when pigs were first provided with enrichment and then the enrichment was taken away. Lesions and scratches can be indicators of aggressive interactions and while these data may be indicative of reduced aggression we did not directly observe aggressive behaviour and acknowledge that other factors may have contributed to the different number of scratches. Combined, these data do indicate that there were several effects of the provision of enrichment blocks and these effects are likely to influence the welfare of pigs.

The benefit(s) of providing enrichment has been evaluated in a number of ways, for example, by evaluating glucocorticoids, the immune system, heart rate variability, agonistic interactions between animals, or changes in the brain [8,13,15]. Our hypothesis was that the benefit of enrichment is not related to the number of interactions that the animal has with the enrichment but whether the provision of enrichment can influence the behaviour of the pigs when tested outside of their home pen. Our data support this hypothesis, and we have evidence that the provision of enrichment to pigs in the sucker phase increased their willingness to interact with their environment and provision of enrichment in the weaner phase may have improved the performance of pigs in the executive function test. Oro-nasal contact with this form of enrichment block has been shown by Winfield et al. [14] to be relatively infrequent before pigs reach about 25 days old, who also found there was a less than 10% probability that pigs would interact with the blocks at this age. Nonetheless, our data indicate that the presence of the enrichment blocks during the sucker phase altered the performance of the pigs in the behavioural tests. An important aspect of environmental enrichment is the opportunity for animals to apply a variety of cognitive processes to solve problems [4] and environments that foster flexible behavioural repertoires are more effective than environments that foster uniformity or are barren [16]. The data from the current experiment suggest that a more complex environment in the sucker phase had benefits for pigs and the benefit was not determined by the number of interactions the pig had with the enrichment. More likely, the benefits for the pig may be that the addition of the blocks provide an opportunity for pigs in the sucker phase to solve problems (albeit simple problems, like how to navigate around or over an enrichment block), and thus exposure to a more complex environment may stimulate the development of a greater behavioural repertoire.

Enrichment in the weaner phase may have assisted in improving the learning ability of the pigs. The proportion of pigs that reached the correct zone in the executive function test was greater and time spent in trap 2 was less for pigs provided with enrichment during the weaner phase than for pigs housed in barren pens during the weaner phase. Similarly, rats housed in enriched environments made less errors in a maze test and displayed greater working memory than rats housed in barren environments [17]. There were accompanying functional changes to the brain of these rats and this indicates that the rats housed in the enriched environment had functional differences in the way their brains had developed [17]. There is little evidence of this in pigs and we present the first, although not conclusive, evidence that enrichment can alter cognitive function in young pigs. It is important to note that previous research in this area used complex enriched environments. The rats housed in enriched pens in the aforementioned research had access to deep litter, toys, running wheels, and an overall more complex environment. The pigs in the current study were provided only with enrichment blocks. Therefore, it is not unexpected that evidence of improved cognition was seen, albeit less compelling and to a lesser extent than previous research in other species. Our results may simply reflect the relative simplicity of the enrichment used in the current experiment.

We have good evidence that pigs younger than 10 weeks of age can learn quickly and learn complex tasks quickly. When data from all pigs were combined and analysed based on performance in successive exposures to the maze test and the executive function test, performance significantly improved over time. For example, the mean time for pigs to solve the maze reduced from 148 s in round 1 to 22 s in round 4. The number of animals that reached the zone with the reward on the first attempt in the executive function test significantly increased from 17% in the first test to 41% in the third test, and the number of zones crossed to reach the reward and the mean time to reach the reward both significantly decreased from test one to test three. Combined, this indicates that the pigs had the ability to learn complex tasks at this age. In particular, the improved performance in the executive function test suggests the pigs learned to differentiate between two audible cues and associate one of those cues with a reward. This is a complex task and the ability of the pigs to learn this task has implications for management practices and housing systems applied at this age. It reaffirms that the sucker and weaner phases are important developmental stages for pigs, and experiences during this time may shape pig behaviour through life.

## 5. Conclusions

The current experiment identified that enrichment in the sucker and weaner phases can affect the behaviour of pigs and their ability to learn. Our data support the notion that the benefits of enrichment cannot be accurately gauged by measuring the interactions the animal has with the enrichments, and it may simply be beneficial to live in a more complex environment. Although this experiment has not identified one clear benefit of the provision of the enrichment blocks, it has identified that enrichment provided in the sucker phase does have benefits, as does enrichment provided in the weaner phase. In addition, pigs in the weaner phase can learn complex tasks. The overall implication of this research is that environmental enrichment likely impacts the behavioural development and learning ability of young pigs. While the current experiment could not identify what the longer-term implications might be, nevertheless we speculate that the pigs provided with enrichment would be better prepared to cope with challenge and thus may adapt faster to new environments. We have highlighted that the early rearing environment is important and that the management and husbandry at an early age can have long-term implications for pigs. The enrichment we used in this study was very simple, an enrichment block that small pigs could move about by rooting/nosing/biting, and we have been able to show a number of effects on the pigs.

## Figures and Tables

**Table 1:** Catalogue of behaviours recorded in the open field and novel object tests.

Behaviour	Description
Emergence	Head and front shoulders cross starting box threshold.
Zone crossed	Head and front shoulders cross over marked lines into a new zone.
Grunt	One low frequency sound produced by the pig or for successive grunting, counted for every 5 s it continued.
Squeal	A high frequency noise produced by the pig.
Urination	Pig expels urine inside the testing arena.
Defecation	Pig expels faeces inside the testing arena.
Jump at wall	Launches body at walls of testing arena.
Interaction	Sniff/softly touch novel object with snout.
Knock	Forceful hit on the novel object (bucket) with the swing of the head.
Avoid	Actively avoids novel object when moving around the arena.

**Table 2:** Catalogue of behaviours recorded in the maze test.

Behaviour	Description
Emergence	Head and front shoulders cross starting box threshold.
Reach reward	Snout touches bowl that contains the reward or engages in eating the reward.
Trap	Head and front shoulders cross over the line marking the entrance of the trap, considered ‘trapped’ until head and shoulders cross back over the entrance line.
Urinate	Piglet expels urine inside the testing arena.
Defecate	Piglet expels faeces inside the testing arena.
Grunt	One low frequency sound produced by the pig or for successive grunting, counted for every 5 s it continued.
Squeal	A high frequency noise produced by the pig.

**Table 3:** Catalogue of behaviours recorded in the executive function test.

Behaviour	Description
Emergence	Head and front shoulders cross starting box threshold.
Zone Entered	Head and front shoulders cross over marked lines into the adjacent zone.
Bowl Reached	Snout touches bowl or cream reward in bowl.
